# SGLT2 inhibitors as a strategy for volume management in peritoneal dialysis: a case series

**DOI:** 10.3389/fneph.2026.1942462

**Published:** 2026-09-29

**Authors:** R. Haridian Sosa Barrios, Miriam Domínguez-Tejedor, Raquel Moreno García, Víctor M. Burguera Vion, Milagros Fernández Lucas, Maite E. Rivera Gorrín

**Affiliations:** 1 Department of Nephrology, Hospital Universitario Ramón y Cajal, Madrid, Spain; 2 Instituto Ramón y Cajal de Investigación Sanitaria (IRYCIS), Madrid, Spain; 3 Universidad de Alcalá (UAH), Madrid, Spain

**Keywords:** peritoneal dialysis, case series, dapagliflozin, encapsulating peritoneal sclerosis, erythropoietin resistance index, fungal peritonitis, SGLT2 inhibitors, ultrafiltration

## Abstract

Sodium–glucose cotransporter 2 inhibitors (SGLT2i) have demonstrated cardiovascular and renal benefits in chronic kidney disease, yet their role in peritoneal dialysis (PD) remains largely unexplored, and clinical experience is limited. We describe, at the individual-patient level, seven consecutive patients treated with an SGLT2i at a single centre (median age 69 years, range 43–73; five men; one with type 2 diabetes; six dapagliflozin, one empagliflozin). The PD prescription was not modified during treatment, and ultrafiltration refers to the 24-hour drained volume. In the four patients in whom the drug was added to established, unchanged PD, ultrafiltration rose in three and fell in one; the single non-responder was also the only low-average transporter and was later transferred to haemodiafiltration. Both patients anuric at baseline increased their ultrafiltration, and in one of them PD was the only feasible modality. Peritoneal transport category was unchanged wherever paired equilibration tests were available. Serum urate fell in the patients with preserved diuresis but not in those who were anuric, whereas the largest ultrafiltration gain occurred in a patient anuric throughout, dissociating the peritoneal response from any renal effect. The erythropoietin resistance index fell in all treated patients, although the magnitude was clinically relevant in a minority and must be read alongside the haemoglobin trajectory. No patient developed anuria *de novo*. One patient died of fungal peritonitis in the context of documented non-adherence, an event reported here with its mechanistic implications. These observations support a direct peritoneal mode of action and warrant prospective evaluation with surveillance for fungal infection.

## Introduction

Peritoneal dialysis (PD) is a home-based renal replacement therapy that offers flexibility and a slower initial decline in residual kidney function compared with haemodialysis. However, fluid overload remains a major complication, affecting up to 25–50% of PD patients and independently predicting cardiovascular mortality (1, 2). Adequate peritoneal ultrafiltration (UF) is essential for volume homeostasis, yet chronic exposure to glucose-based dialysate paradoxically damages the peritoneal membrane through glucose degradation products, oxidative stress and fibrosis, ultimately leading to UF failure (3).

Sodium–glucose cotransporter 2 inhibitors (SGLT2i) block glucose and sodium reabsorption in the proximal tubule, inducing glucosuria, osmotic diuresis and tubuloglomerular feedback–mediated reduction in intraglomerular pressure (4). Landmark trials have demonstrated benefits across a broad range of kidney function (5–7). Additionally, SGLT2i stimulate erythropoiesis via hypoxia-inducible factor 2α upregulation, consistently increasing haemoglobin in randomized trials (8).

Translational studies have shown that SGLT2 is expressed in the human peritoneum (9), that its inhibition reduces dialysate glucose absorption in animal models (10) and that it mitigates peritoneal fibrosis (11). However, clinical data in PD remain scarce (12–16): no placebo-controlled trial has reported results, although the EMPOWERED trial protocol has been published (17), and a recent review confirmed the paucity of outcome data (18). The rationale for using these agents in PD is threefold. Their cardiovascular and metabolic actions do not depend on renal clearance and need not be forfeited when residual function is exhausted; the peritoneum expresses SGLT2, which offers a plausible target for reducing dialysate glucose absorption and preserving the osmotic gradient (9, 10); and for patients with ultrafiltration failure and no alternative modality there is at present no other pharmacological option.

We therefore describe the systematic use of these agents in an unselected, predominantly non-diabetic PD cohort. Given the sample size, the purpose of this report is descriptive: to document what happened to seven patients, individually and without inference, in clinical scenarios for which no evidence currently exists.

## Methods

### Design and setting

Retrospective, single-centre, descriptive case series comprising all consecutive patients receiving PD at Hospital Universitario Ramón y Cajal in whom an SGLT2i was prescribed between February 2023 and March 2025. No patient meeting this criterion was excluded.

### Ethics

The study was approved by the Clinical Research Ethics Committee of Hospital Universitario Ramón y Cajal (reference 188/26) and conducted in accordance with the Declaration of Helsinki and Spanish Law 14/2007 on Biomedical Research. Given the retrospective design and the use of fully anonymized data derived from routine care, the Committee waived the requirement for informed consent for participation.

### Treatment decision and off-label framework

All prescriptions were made outside the authorised conditions of use (19, 20), under the Spanish regulatory framework for medicines used in special situations (Royal Decree 1015/2009). Each decision was taken collectively in the Peritoneal Dialysis Unit clinical session. The reasoning differed by indication: recurrent congestive admissions in two patients with type 2 cardiorenal syndrome, in whom the heart failure indication specifies no lower renal threshold for continuation; insufficient peritoneal ultrafiltration with no conventional alternative in three; preservation of residual kidney function in one; and adjuvant therapy in one patient with histologically confirmed encapsulating peritoneal sclerosis (11). In the anuric patients no renal effect could be expected and the rationale was exclusively peritoneal (9).

### Timing of treatment

The interval between the start of PD and the start of the SGLT2i is reported for every patient in **Table 1**. Four patients began therapy on established PD, two began before PD initiation and one began after prolonged PD vintage. One patient had received the drug in advanced CKD, had it withdrawn following an episode of acute kidney injury, and resumed it before starting PD.

**Table 1:** Baseline characteristics, timing of treatment and peritoneal transport, by patient (N = 7).

Pt	Age	Sex	Cause of CKD	DM	Agent	PD start	SGLT2i start	Timing vs PD start	Indication	Transport pre/post	Urine (mL/24h)	UF base
1	70	M	Cardiorenal syndrome type 2	Yes	Empagliflozin	25/10/2022	21/02/2023	3.9 mo after	Heart failure/volume	High-avg/High-avg	1800	500
2	73	F	Cortical necrosis	No	Dapagliflozin	11/04/2023	14/08/2024	16.1 mo after	Increase peritoneal UF	Low-avg/n.a.	590	800
3	52	M	Chronic tubulointerstitial nephritis	No	Dapagliflozin	11/11/2024	05/12/2024	0.8 mo after	Increase peritoneal UF	n.a./High-avg	130	660
4	43	F	Lupus nephritis	No	Dapagliflozin	17/07/2024	06/03/2025	7.6 mo after	Increase peritoneal UF	Average/Average	0	658
5	71	M	Membranous nephropathy	No	Dapagliflozin	06/05/2024	10/05/2023	11.9 mo before	Preserve residual function	Average/Average	2900	n.a.
6	69	M	Cardiorenal syndrome type 2	No	Dapagliflozin	06/05/2024	05/02/2024	3.0 mo before	Heart failure/volume	Low-avg/Low-avg	1300	n.a.
7	66	M	Long-standing CKD with acquired cirrhosis (cardio-hepato-renal)	No	Dapagliflozin	20/10/2021	23/04/2024	30.1 mo after	Adjuvant in EPS	High-avg/n.a.	1690	n.a.

Icodextrin was used in all patients except Patient 5. Patient 6 had received dapagliflozin in advanced CKD from 17/05/2022 to 10/04/2023, withdrawn for acute kidney injury and restarted on 05/02/2024. UF, 24-hour peritoneal ultrafiltration (mL/24h); EPS, encapsulating peritoneal sclerosis; n.a., not available.

### Peritoneal dialysis prescription

The PD prescription — modality, number and duration of dwells, glucose concentration and use of icodextrin — was not modified during the treatment period in any patient. Ultrafiltration refers to the total 24-hour drained volume minus the volume infused, recorded by the patient at home. Icodextrin-based solutions were used in six patients.

### Definitions and measurements

Residual kidney function was determined from 24-hour urine collections and is expressed as the arithmetic mean of the renal urea and creatinine clearances, normalized to body surface area. No creatinine-based estimating equation was used at any point, and the term estimated glomerular filtration rate is not applicable to this cohort. Anuria was defined as a urine output below 200 mL/24h. Peritoneal transport status was categorised by peritoneal equilibration test. Doses of erythropoiesis-stimulating agents were unified using the conversion 1 µg darbepoetin alfa = 200 IU epoetin alfa, and the erythropoietin resistance index was calculated as the weekly equivalent dose divided by haemoglobin.

### Analysis

Analysis is descriptive only: continuous variables as median and range, categorical variables as counts and percentages. No hypothesis testing was performed, being neither justified nor informative at this sample size; every outcome is presented as an individual trajectory.

## Results

### Cohort

Seven patients on PD treated with SGLT2i were enrolled at a single university center between February 2023 and March 2025 (**Table 1**). The median age was 69 years (range 43–73). Five patients (71.4%) were male. Only one (14.3%) had type 2 diabetes mellitus, and this patient was also the only one treated with empagliflozin. CKD etiologies were heterogeneous: cardiorenal syndrome type 2 (n = 2), long-standing CKD with subsequently acquired cirrhosis and cardio-hepato-renal syndrome (n = 1), lupus nephritis (n = 1), membranous nephropathy (n = 1), chronic tubulointerstitial nephritis (n = 1), and cortical necrosis (n = 1). Four patients started the SGLT2i on established PD, at a median of 5.8 months (range 0.8–16.1) after starting the technique; one started after 30 months on PD; and two started before PD initiation, 11.9 and 3.0 months respectively. Six patients received dapagliflozin 10 mg/day and one received empagliflozin 10 mg/day. Six patients (85.7%) used icodextrin-based solutions. Two patients (28.6%) were anuric at baseline by the prespecified definition.

### Peritoneal ultrafiltration

Paired baseline and follow-up UF data were available for four patients (Patients 1–4), who are precisely the four in whom the drug was introduced onto an established and unchanged PD prescription. Patients 5 and 6 have no baseline value because they were already receiving the drug when the technique began, and Patient 7 has no ultrafiltration data at all. Median UF rose from 659 mL/24h (range 500–800) at baseline to 988 (758–1139) at one month and 1250 (721–1596) at three months (**Figure 1A**). Three of the four patients increased their ultrafiltration, by 1096, 842 and 340 mL/24h at three months; one decreased it by 79 mL/24h. Patient 2 was the sole non-responder, and was also the only low-average transporter in the group; he was transferred to haemodiafiltration three months after starting therapy because of pulmonary hypertension with intractable volume management. In Patients 3 and 4 (near-anuric and anuric, respectively), SGLT2i-enhanced UF was critical for sustaining PD viability. Peritoneal transport category was unchanged between paired equilibration tests in all four patients in whom these were available. Clinically, peripheral oedema was present in four patients at baseline and in two at six months. Thus, patient number 1, who had suffered from frequent episodes of cardiac failure, notably improved following UF increase after SGLT2i. In addition, peritoneal dialysis was the only possible treatment for patient number 3. All vascular accesses were exhausted, the inferior vena cava was filiform, and kidney transplantation was not an option. Increasing ultrafiltration with SGLT2 inhibitors was essential to maintain euvolemia in this anuric patient.

**Figure 1:**
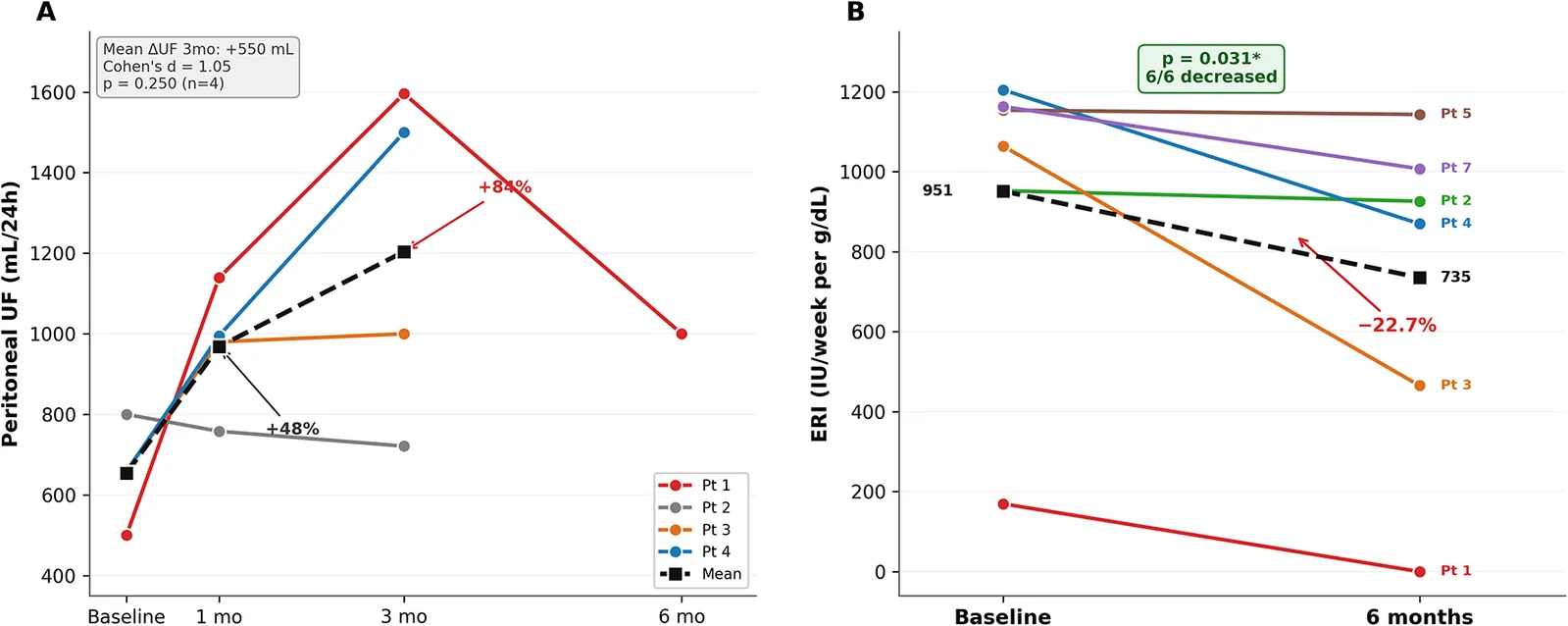
Individual outcome trajectories. **(A)** Individual peritoneal UF trajectories in the four patients with paired baseline data. **(B)** Paired erythropoietin resistance index (ERI) at baseline and 6 months in the six ESA-treated patients. ERI decreased in all patients, with the magnitude of change varying widely between individuals. Conversion factor: 1 µg darbepoetin = 200 IU epoetin. Cohort mean lines and all statistical annotations have been removed from both panels.

### Residual kidney function and diuresis

Residual kidney function was available in five patients at baseline and in four throughout. In the four with complete data the median value was 10.6 mL/min at baseline, 10.5 at one month and 14.5 at six months; two showed a transient fall and recovery, one remained stable and one rose progressively. The fall in one patient coincided with a simultaneous fall in Kt/V and reflected an intercurrent clinical event. Both patients anuric at six months were already anuric at baseline, and no patient developed anuria *de novo*. Among the five with preserved diuresis, urine output decreased in three (by 200, 210 and 400 mL/24h), was unchanged in one and increased in one. Three individual Kt/V values fell below 1.7 during follow-up (**Table 2**). The urinary protein-to-creatinine ratio, available in five patients, decreased in three (by 24%, 42% and 58%) and increased in two.

**Table 2:** Volume status and kidney function by patient.

Pt	UF base	UF 1 mo	UF 3 mo	UF 6 mo	Urine base	Urine 1 mo	Urine 6 mo	RKF base	RKF 1 mo	RKF 6 mo	Kt/V base	Kt/V 1 mo	Kt/V 6 mo
1	500	1139	1596	1000	1800	2200	1600	11.7	2.4	8.8	1.92	1.20	1.76
2	800	758	721	n.a.	590	400	380	1.2	0.6	n.a.	1.94	1.89	n.a.
3	660	980	1000	n.a.	130	0	0	n.a.	n.a.	n.a.	n.a.	n.a.	n.a.
4	658	995	1500	n.a.	0	0	0	n.a.	n.a.	n.a.	1.97	1.95	2.30
5	n.a.	390	450	400	2900	2700	2900	9.5	10.2	8.5	1.63	1.85	1.74
6	n.a.	1135	1000	1400	1300	1200	900	24.9	12.7	21.7	3.33	3.29	1.44
7	n.a.	n.a.	n.a.	n.a.	1690	2000	2000	9.2	10.8	20.2	4.41	n.a.	n.a.

UF, 24-hour peritoneal ultrafiltration (mL/24h); RKF, residual kidney function from 24-hour urine collection (mL/min/1.73 m²); n.a., not available. The PD prescription was unchanged throughout. Anuria was defined as urine output below 200 mL/24h.

### Erythropoietin resistance and anaemia

Six patients were receiving ESA at baseline (four epoetin alfa, two darbepoetin alfa). Doses were unified using the conversion 1 µg darbepoetin = 200 IU epoetin. The ERI decreased in all six patients (**Figure 1B**) although the magnitude of change varied widely and was clinically relevant in only three. Responses ranged from complete ESA withdrawal (Patient 1, whose haemoglobin rose from 11.8 to 15.3 g/dL) to changes below three per cent in two patients. In one patient the index improved only because the dose was reduced by 60% while haemoglobin fell from 9.4 to 8.6 g/dL, which does not represent improved responsiveness. In another the direction of change depends entirely on the conversion factor, because the agent was switched during follow-up. The clearest response was that of Patient 7, whose haemoglobin rose from 8.6 to 9.9 g/dL on an unchanged dose. Median haemoglobin for the cohort rose from 10.4 to 10.8 g/dL, and individual values are given in **Table 3**.

**Table 3:** Anaemia, metabolic parameters and clinical outcome by patient.

Pt	Hb base	Hb 6 mo	ESA base	ESA 6 mo	ERI base	ERI 6 mo	Urate base	Urate 6 mo	P/Cr base	P/Cr 6 mo	Clinical outcome
1	11.8	15.3	Epoetin 2000 IU/wk	Withdrawn (Jan 2024)	169	0	7.7	5.9	1570	657	Died — refractory heart failure
2	10.5	10.8	Epoetin 10–000 IU/wk	Epoetin 10–000 IU/wk	952	926	5.1	3.3	1555	n.a.	Technique failure — transferred to HDF, Nov 2024
3	9.4	8.6	Epoetin 10–000 IU/wk	Epoetin 4000 IU/wk	1064	465	6.0	6.0	n.a.	n.a.	Died May 2025 — fungal peritonitis, documented non-adherence
4	9.3	11.5	Darbepoetin 40 µg/5 d	Epoetin 10–000 IU/wk	1204	870	6.6	7.0	n.a.	n.a.	Alive, continuing PD
5	10.4	10.5	Darbepoetin 60 µg/wk	Darbepoetin 60 µg/wk	1154	1143	6.5	5.6	2701	1564	Alive, continuing PD
6	14.1	14.0	None	None	n.a.	n.a.	11.7	5.1	153	264	Died — refractory heart failure
7	8.6	9.9	Epoetin 10–000 IU/wk	Epoetin 10–000 IU/wk	1163	1007	9.1	8.0	311	235	PD discontinued Jan 2025; alive in CKD stage 4 without RRT

Hb, haemoglobin (g/dL); ESA, erythropoiesis-stimulating agent; ERI, erythropoietin resistance index (weekly epoetin-equivalent IU per g/dL), calculated with the conversion 1 µg darbepoetin alfa = 200 IU epoetin alfa; urate in mg/dL; P/Cr, urinary protein-to-creatinine ratio (mg/g); HDF, haemodiafiltration; RRT, renal replacement therapy; n.a., not available.

### Serum urate

Serum urate fell in the five patients with preserved diuresis, by between 0.9 and 6.6 mg/dL. It was unchanged in the patient who became anuric during the first month and rose, from 6.6 to 7.0 mg/dL, in the only patient anuric throughout the entire follow-up. That same patient showed the largest sustained increase in ultrafiltration of the series.

### Safety and clinical outcomes

No hypoglycaemia, euglycaemic ketoacidosis or genital mycotic infection was recorded. One patient required temporary withdrawal for acute kidney injury, with uneventful resumption; this episode occurred before PD initiation. Three patients died during follow-up: two from end-stage cardiac disease in the context of type 2 cardiorenal syndrome, and the third on 31 May 2025 from fungal peritonitis, after approximately six months of dapagliflozin. In this patient, for whom PD was the only available modality, the peritonitis was treated according to the fungogram, but he was persistently non-adherent, subsequently declined treatment and did not attend appointments, and the infection recurred in that setting.

### Encapsulating peritoneal sclerosis (patient 7)

This patient started PD in October 2021 for long-standing chronic kidney disease and developed cirrhosis subsequently; the terminal picture was a cardio-hepato-renal syndrome rather than hepatorenal syndrome as the cause of kidney failure. Encapsulating peritoneal sclerosis was diagnosed in April 2024, after approximately 30 months on the technique, and confirmed histologically on surgical specimens. Dapagliflozin was started as adjuvant therapy at that time. Residual kidney function improved progressively and PD was discontinued in January 2025, after nine months of treatment. This did not represent recovery from acute kidney injury nor resolution of chronic kidney disease: the patient remained in stage 4 and has continued without renal replacement therapy since. No ultrafiltration data are reported for him, since in cirrhosis with ascites the drained volume does not equate to ultrafiltration.

## Discussion

This case series provides real-worldobservations on the use of SGLT2i in an unselected, predominantly non-diabetic PD population. Its value is clinical and mechanistic rather than statistical: it documents outcomes in situations — anuria without alternative modality, recurrent congestive admissions, encapsulating peritoneal sclerosis — in which the PD community currently improvises without evidence.

Ultrafiltration increased substantially in three of the four patients in whom the drug was added to an unchanged PD prescription, consistent with the documented expression of SGLT2 in human peritoneal biopsies (9). Because the prescription was not modified, the obvious alternative explanation — a higher dialysate glucose concentration or additional dwells — is excluded by design. We make no claim of statistical significance: with four patients none could be supported, and the sample-size assumptions of the ongoing EMPOWERED trial (17) indicate that a larger cohort will be needed to quantify this effect. Our observations are directionally consistent with previous reports in PD (12–14) and with real-world data on anaemia and residual kidney function (15).

### The strongest mechanistic argument in this series arises from a dissociation observed within a single patient

Serum urate fell only in those with preserved diuresis and rose in the patient anuric throughout, as the physiology predicts, since the uricosuric action of these agents requires urine flow. The anuric patients therefore act as an internal control confirming that the renal route behaved as expected. Yet that same patient, in whom no renal drug effect was demonstrable, showed the largest sustained ultrafiltration gain of the series. A response present in the peritoneum and absent in the kidney, in the same individual at the same time, argues for a direct peritoneal mechanism. That peritoneal transport category did not change in any patient with paired equilibration tests points the same way, suggesting improved osmotic conduction — slower dissipation of the glucose gradient (3, 10) — rather than altered small-solute permeability.

### Transport phenotype may condition the response

The three responders were average or high-average transporters, whereas the single non-responder was the only low-average transporter, the phenotype with the least baseline peritoneal glucose absorption and therefore the least to gain from blocking it. With four patients this is a hypothesis, but one that deserves prespecified stratification in prospective trials.

The erythropoietin resistance index fell in every treated patient, which is consistent with the HIF-2α–mediated erythropoietic effect of these agents (8). The magnitude, however, was clinically relevant in a minority, and the index must be interpreted alongside the haemoglobin trajectory: a fall driven by dose reduction in a patient whose anaemia worsened is not improved responsiveness. The absence of systematic iron and inflammatory data is a material limitation for any statement about erythropoietin responsiveness.

### A safety observation requires emphasis

One patient died of fungal peritonitis while receiving dapagliflozin. Causality cannot be inferred from a single case in a non-adherent patient who declined treatment, and fungal peritonitis in PD is classically associated with prior antibiotic exposure. Nevertheless, the mechanism we propose for the ultrafiltration benefit — reduced peritoneal glucose absorption through inhibition of mesothelial SGLT2 — necessarily implies a higher residual intraperitoneal glucose concentration for a longer dwell period. This is a biologically plausible route to increased fungal risk, and it has not to our knowledge been raised previously. In a field where this off-label use is spreading ahead of the evidence, we consider it necessary to state the hypothesis and to recommend explicit surveillance for fungal peritonitis in prospective studies.

We deliberately refrain from drawing an analogy with the haemodynamic pattern described in DAPA-CKD and EMPA-KIDNEY. On restricted analysis of the patients with complete paired data, the apparent early fall in residual kidney function was generated by a single patient with an intercurrent clinical event, and by median values no such fall existed. Extrapolating a tubuloglomerular feedback pattern to patients with minimal residual function and concurrent peritoneal clearance is not supportable with these data.

The absence of drug-attributable adverse events than the event discussed above is consistent with pharmacokinetic data confirming no dapagliflozin accumulation in PD (16).

### Limitations

This study has several important limitations. The very small sample size (n = 7) precludes any inferential conclusion, and no hypothesis testing was therefore attempted; the observations are hypothesis-generating only. Paired ultrafiltration data were available in four patients. The retrospective, single-center design without a control group prevents causal inference. ESA heterogeneity necessitated dose conversion with a standard factor that has known inter-individual variability, and in one patient the direction of change in the resistance index depends on that factor. Follow-up duration and data completeness varied across patients. One patient began therapy three weeks after starting PD, so his baseline ultrafiltration is that of an incident patient. Finally, the single diabetic patient is also the only one given empagliflozin, the largest ultrafiltration responder and the only case of ESA withdrawal, so both principal observations derive disproportionately from the least representative patient; in a diabetic, glycosuria-driven osmotic diuresis and improved glycaemic control confound both outcomes. The mortality observed, although largely attributable to underlying disease, limits long-term outcome assessment.

## Conclusion

SGLT2 inhibition augmented peritoneal ultrafiltration in patients receiving an unchanged dialysis prescription, and did so in the absence of any demonstrable renal effect, which places the site of action in the peritoneum rather than the kidney. In patients without residual function and without alternative modality, this gain was the difference between a viable technique and none. Erythropoietin requirements fell consistently in direction though not uniformly in magnitude, an effect that does not appear to require preserved renal parenchyma. Against this stands a fungal peritonitis whose plausibility follows from the very process that confers the ultrafiltration benefit, and which must be actively sought rather than assumed absent. Prospective controlled evaluation should stratify by peritoneal transport phenotype and monitor infectious outcomes explicitly.

## Data Availability

The original contributions presented in the study are included in the article/**Supplementary Material**. Further inquiries can be directed to the corresponding author.
