# Supplementary material for: SGLT2 inhibitors as a strategy for volume management in peritoneal dialysis: a case series
Source: Front Nephrol. 2026 Sep 29;6:1942462. doi: 10.3389/fneph.2026.1942462 (PMC13657984; doi:10.3389/fneph.2026.1942462)
Supplement: Supplementary file 1 [file Table1.docx]

**SUPPLEMENTARY MATERIAL**

**This document contains three supplementary tables referenced in the main manuscript:**

**Table S1.** Individual peritoneal ultrafiltration trajectories and ΔUF analysis (7 patients, 4 time points).

**Table S2.** Individual ESA dose evolution, hemoglobin response, and ERI calculations (7 patients).

**Table S3.** Summary of published clinical and preclinical studies on SGLT2 inhibitors in dialysis (7 clinical + 4 preclinical), with critical synthesis.

**Table S1. Individual Peritoneal Ultrafiltration Trajectories and ΔUF Analysis**

| **Pt** | **CKD Etiology** | **UF Baseline** | **UF 1 mo** | **ΔUF 1 mo** | **UF 3 mo** | **ΔUF 3 mo** | **UF 6 mo** | **ΔUF 6 mo** | **Anuric** | **Clinical Interpretation** |
| --- | --- | --- | --- | --- | --- | --- | --- | --- | --- | --- |
| 1 | CRS type 2 | 500 | 1139 | **+639 (+128%)** | 1596 | **+1096 (+219%)** | 1000 | **+500 (+100%)** | No | Strongest responder; HF patient with no further readmissions after SGLT2i; UF decline at 6 mo may reflect membrane fatigue |
| 2 | Cortical necrosis | 800 | 758 | **−42 (−5%)** | 721 | **−79 (−10%)** | N/A | **N/A** | No | Only non-responder; high-average transporter (MA); lost to follow-up at 6 mo |
| 3 | NTIC | 660 | 980 | **+320 (+49%)** | 1000 | **+340 (+52%)** | N/A | **N/A** | Near | Near-anuric (130 mL/d); became anuric; SGLT2i-enhanced UF critical for PD viability; died (progressive CKD) |
| 4 | Lupus nephritis | 658 | 995 | **+337 (+51%)** | 1500 | **+842 (+128%)** | N/A | **N/A** | Yes | Anuric; largest sustained response; UF sole mechanism for fluid removal; average transporter |
| 5 | Membranous NP | N/A | 390 | **N/A** | 450 | **N/A** | 400 | **N/A** | No | No baseline UF (started PD + SGLT2i concurrently); stable UF throughout |
| 6 | CRS type 2 | N/A | 1135 | **N/A** | 1000 | **N/A** | 1400 | **N/A** | No | No baseline UF (started PD + SGLT2i concurrently); good UF; died (refractory HF at 16 mo) |
| 7 | SHR / EPS | N/A | N/A | **N/A** | N/A | **N/A** | N/A | **N/A** | No | EPS with no UF data; eGFR improved 9.2→23.9 mL/min; withdrawn from PD at 10 mo |
|  | **Mean (n=4 paired)** | **654±123** | **968±157** | **+314±279** | **1204±415** | **+550±524** | – | – |  | **Wilcoxon p = 0.250; Cohen’s d = 1.05 (3 mo); 75% responders** |

*Values in mL/24h. ΔUF = UF post − UF baseline. Green cells = positive ΔUF; yellow cells = negative ΔUF. Blue row = summary statistics for the 4 patients with paired data. Patients 5–7 lacked baseline UF measurements and are excluded from paired analyses. CRS = cardiorenal syndrome; NTIC = chronic tubulointerstitial nephritis; NP = nephropathy; SHR = hepatorenal syndrome; EPS = encapsulating peritoneal sclerosis; HF = heart failure.*

**Table S2. Individual ESA Dose Evolution, Hemoglobin Response, and ERI Calculations**

| **Pt** | **CKD Etiology** | **ESA Baseline (Agent + Dose)** | **ESA 6 Months (Agent + Dose)** | **EPO eq. PRE (IU/wk)** | **EPO eq. POST (IU/wk)** | **Hb PRE (g/dL)** | **Hb 6 mo (g/dL)** | **ERI PRE** | **ERI POST** | **Interpretation** |
| --- | --- | --- | --- | --- | --- | --- | --- | --- | --- | --- |
| 1 | CRS-2 | Binocrit 2,000 IU/wk | Discontinued | 2,000 | 0 | 11.8 | 15.3 | 169.5 | 0.0 | ESA withdrawn; Hb ↑ 3.5 g/dL; ERI ↓100% |
| 2 | Cort. necrosis | Binocrit 10,000 IU/wk | Binocrit 10,000 IU/wk | 10,000 | 10,000 | 10.5 | 10.8 | 952.4 | 925.9 | Stable dose; Hb stable; ERI −2.8% |
| 3 | NTIC | Binocrit 10,000 IU/wk | Binocrit 4,000 IU/wk | 10,000 | 4,000 | 9.4 | 8.6 | 1063.8 | 465.1 | 60% dose reduction; ERI −56.3% |
| 4 | LN | Aranesp 40 µg q5d (= 56 µg/wk) | Binocrit 10,000 IU/wk | 11,200* | 10,000 | 9.3 | 11.5 | 1204.3 | 869.6 | Agent switch; Hb ↑ 2.2; ERI −27.8% |
| 5 | MN | Aranesp 60 µg/wk | Aranesp 60 µg/wk | 12,000* | 12,000* | 10.4 | 10.5 | 1153.8 | 1142.9 | Stable dose; Hb stable; ERI −0.9% |
| 6 | CRS-2 | None | None | 0 | 0 | 14.1 | 14.0 | N/A | N/A | No ESA; excluded from ERI analysis |
| 7 | SHR/EPS | Binocrit 10,000 IU/wk | Binocrit 10,000 IU/wk | 10,000 | 10,000 | 8.6 | 9.9 | 1162.8 | 1007.0 | Stable dose; Hb ↑ 1.3; ERI −13.4% |
|  | **Mean ± SD (n=6)** |  |  | **9,200** | **7,667** | **10.0** | **10.9** | **951±359** | **735±389** | **6/6 decreased; Wilcoxon p = 0.031; ΔERI −216±202 (−22.7%)** |

*ESA = erythropoiesis-stimulating agent. EPO eq. = weekly equivalent EPO dose (IU/week). Conversion factor: 1 µg Aranesp (darbepoetin alfa) = 200 IU Binocrit (epoetin alfa). *Converted from darbepoetin. For Patient 4: Aranesp 40 µg every 5 days = 40 × (7/5) = 56 µg/week = 11,200 IU/week equivalent. ERI = weekly EPO equivalent dose (IU) / hemoglobin (g/dL). Patient 6 excluded from ERI analysis (no ESA at any time point). Blue row = summary statistics. CRS = cardiorenal syndrome; NTIC = chronic tubulointerstitial nephritis; LN = lupus nephritis; MN = membranous nephropathy; SHR = hepatorenal syndrome; EPS = encapsulating peritoneal sclerosis.*

**Table S3. Summary of Published Clinical and Preclinical Studies on SGLT2 Inhibitors in Dialysis**

| **Study** | **N** | **Drug** | **Design** | **Primary Endpoints** | **Key Results** |
| --- | --- | --- | --- | --- | --- |
| **CLINICAL STUDIES IN PERITONEAL DIALYSIS** | | | | | |
| **Alhwiesh et al., 2022** | 50 | Dapagliflozin 10 mg/d | Prospective cohort T2DM on APD 12 months | PET transport status; metabolic parameters; UF volume | UF ↑ 230 mL/24h; diuresis ↑ 253 mL/24h; HbA1c ↓ 1.6%; weight ↓ 2.6 kg; SBP ↓ 14 mmHg; CRP ↓; PET unchanged; no serious AEs |
| **Lai et al., 2023** | 4 | Dapagliflozin 10 mg/d | Case report incident PD 5–85 days | UF volume; blood pressure | UF ↑ in all 4 patients; BP improved; all fast transporters; no AEs |
| **Lai et al., 2024** | 4 | Dapagliflozin 10 mg/d | Follow-up of 2023 case report | UF volume; residual diuresis | UF and diuresis maintained; BP stable; no AEs |
| **Moral Berrio / De La Flor et al., 2024** | 16 | Empagliflozin (13) Dapagliflozin (3) | Retrospective single-center DM + non-DM 6 months | RKF preservation (KrU, ClCr, residual diuresis) | RKF preserved at 6 mo; diuresis maintained; trend ↓ proteinuria and ↓ BP; 2 minor AEs (DM group) |
| **PHARMACOKINETIC STUDY** | | | | | |
| **Barreto et al., 2023** | 6 PD +6 HD +6 ctrl | Dapagliflozin 10 mg single | PK study open-label | Pharmacokinetics: Cmax, AUC, t½ | Not dialyzable; no accumulation; Cmax similar across groups; well tolerated |
| **CLINICAL STUDY IN INCREMENTAL HEMODIALYSIS (CONTEXTUAL)** | | | | | |
| **De La Flor et al., 2023** | 7 | SGLT2i (various) | Retrospective T2DM on iHD 12 months | RKF preservation (KrU) | KrU ↑ 4.91→7.28 mL/min (p=0.028); HbA1c, uric acid, proteinuria ↓; safe in incremental HD |
| **TRIAL PROTOCOL (RESULTS PENDING)** | | | | | |
| **Doi et al., 2024** | 30 (plan) | Empagliflozin 10 mg/d | RCT protocol double-blind crossover | Daily UF volume; residual renal function | EMPOWERED; results pending; first placebo-controlled RCT in PD |
| **PRECLINICAL AND TRANSLATIONAL STUDIES** | | | | | |
| **Schricker et al., 2022** | – | N/A (expression) | Human peritoneal biopsies (PD vs non-PD) | SGLT2 expression in peritoneum | SGLT2 expressed in human peritoneum; upregulated in long-term PD; GLUT1/GLUT3 also expressed |
| **Zhou et al., 2019** | – | Empagliflozin | Rat PD model + human PMCs | Glucose absorption from PD solution | SGLT2i suppressed glucose uptake by mesothelial cells; reduced glucose absorption; increased UF in rat model |
| **Balzer et al., 2020** | – | Dapagliflozin (intraperitoneal) | Mouse model chronic high-glucose PD | Peritoneal fibrosis and UF failure | IP dapagliflozin mitigated fibrosis; reduced submesothelial thickening; attenuated TGF-β/Smad; improved UF capacity |
| **Martus et al., 2023** | – | Phlorizin (dual SGLT1/2) | Rat PD model 120-min dwell | Glucose transport across peritoneum | Reduced glucose diffusion at 60 min; no difference solute/water at 120 min; suggests time-dependent route |

*APD = automated peritoneal dialysis; iHD = incremental hemodialysis; PK = pharmacokinetics; RCT = randomized controlled trial; RKF = residual kidney function; PET = peritoneal equilibration test; PMCs = peritoneal mesothelial cells; TGF-β = transforming growth factor beta; IP = intraperitoneal; T2DM = type 2 diabetes mellitus; UF = ultrafiltration; AEs = adverse events.*
